# Sensitivity and Specificity Analysis of Fine Needle Aspiration Cytology (FNAC) for Thyroid Swelling

**DOI:** 10.7759/cureus.54913

**Published:** 2024-02-26

**Authors:** Mangesh G Kohale, Anupama V Dhobale, Nandkishor J Bankar, Neha Bhatt, Akshay H Salgar

**Affiliations:** 1 Pathology, Datta Meghe Medical College, Datta Meghe Institute of Higher Education and Research (DU), Nagpur, IND; 2 Obstetrics and Gynaecology, Datta Meghe Medical College, Datta Meghe Institute of Higher Education and Research (DU), Nagpur, IND; 3 Microbiology, Jawaharlal Nehru Medical College, Datta Meghe Institute of Higher Education and Research (DU), Wardha, IND; 4 Pathology, Dr. Rajendra Gode Medical College, Amravati, IND; 5 Preventive Medicine, Government Medical College and Hospital, Baramati, IND

**Keywords:** tumor, goitre, fnac, specificity, sensitivity

## Abstract

Introduction

Thyroid gland disorders, such as goiters or tumor masses, are the result of localized or systemic aberrations of the thyroid gland. The purpose of this research was to see how effective fine needle aspiration cytology (FNAC) was in detecting thyroid swelling in patients with thyroid swelling. It is critical to be able to differentiate between benign and malignant thyroid nodules to reduce unnecessary thyroid surgeries. It is hypothesized that FNAC is not a reliable diagnostic tool to detect thyroid nodules in patients with thyroid swelling and that there is a significant variation between the number of thyroid nodules detected by FNAC and the number of thyroid nodules that were eventually diagnosed as malignant by surgery. The significance of this research shows the effectiveness of diagnostic tests for thyroid nodules in patients with thyroid swelling can help reduce unnecessary surgeries and improve patient care.

Methods

This cross-sectional study was carried out at the Department of Pathology at a tertiary care hospital in central India, from March 1, 2022 to June 31, 2022. Fifty patients with thyroid swelling were covered in the study. All patients were chosen after an ultrasound revealed goiter on clinical grounds.

Results

The majority of patients in the age group 31-40 years (33.33%) were female (74.67%). About 54.67% of the patients had a single thyroid nodule. FNAC had a sensitivity of 95.38%, a specificity of 53.33%, a positive predictive value of 86.67% and a negative predictive value of 88.57%.

Conclusion

FNAC is a simple, economical, and commonly used first-line diagnostic method for thyroid cancer. A false negative or false positive cytological diagnosis may be produced as a result of thyroid cytology. The study emphasizes the need to improve basic healthcare in rural India by treating FNAC as a first-line diagnostic test for thyroid swellings to guide management, although it does not replace histopathology.

## Introduction

Thyroid gland disorders, such as goiters or tumor masses, are the result of localized or systemic aberrations of the thyroid gland. After diabetes mellitus, the thyroid gland is the organ most often responsible for endocrine problems. The most common cause of thyroid dysfunction is the development of autoimmune antibodies against the thyroid (Hashimoto thyroiditis), and it is estimated to affect up to 5% of adults. Other causes of thyroid dysfunction include toxins, diet, stress, and certain medications. Thyroid dysfunction can cause a wide variety of symptoms, including weight gain, fatigue, hair loss, muscle weakness, depression, and feeling cold [1]. Thyroid enlargement is the most common cause of neck swelling, but there can also be other differential diagnosis like sebaceous cyst, lipoma, lymphangiomas, dermoids, thyroglossal cysts, pleomorphic adenoma, Warthin tumour and reactive neck lymphadenopathy [2]. When swelling is not caused by thyroid enlargement, proper diagnosis and treatment becomes difficult. There may also be a variety of pressure symptoms affecting the trachea, esophagus, and major blood vessels, in addition to cosmetic deformities, resulting from swelling of the neck according to size and histological type. A biopsy may occasionally be required to diagnose cancer, particularly in the case of adenomas [3].

Despite significant advances in our understanding of thyroid tumors, there are still issues and unanswered questions. The first-line diagnostic technique to assess thyroid nodules and diffuse thyroid lesions is fine needle aspiration cytology (FNAC) of the thyroid gland. It is a method used to verify benign lesions and prevent unnecessary surgery [4,5]. The current study aimed to examine the sensitivity and specificity in thyroid enlargement.

## Materials and methods

The present cross-sectional study was conducted at Datta Meghe Medical College and Shalinitai Meghe Hospital and Research Center (DMMC & SMHRC) in central India (IRB No - DMMC (DU)/IEC/2022/78).

Fifty patients with swelling of the thyroid gland were included in the study from March 1, 2022, to June 31, 2022. To establish the sample size, a proportion of disease and a marginal error were calculated using the formula n = z2pq / d2. In order to establish the sample size, z 1.96 (95% confidence interval and 80% error rate) was used, q=1-p (1-p represents the probability of concordance) and d=marginal error.

Subjects who met the inclusion criteria of thyroid gland swelling were selected for the study. Patients with end-stage renal failure, all neck lesions with a history of diagnosis, cases with histological confirmation, and patients who had undergone surgery were excluded. A thorough history was taken, paying special attention to discomfort, dysphagia, duration of lump, hoarseness, and fever. A complete general and systemic physical examination was completed. During the local inspection of the swelling, the following characteristics were noted: size, number, position, consistency, shape, translucency, compressibility, adherence to the underlying tissues, swaying during deglutition, tongue protrusion, and any accompanying lymphadenopathy.

Relevant laboratory tests such as the Mantoux test, erythrocyte sedimentation rate (ESR), and thyroid function tests were performed. Thyroid aspiration is best performed with 22 or 23-gauge needles by an experienced operator, preferably a cytopathologist. The use of larger caliber needles is not recommended as they result in bloody aspirates. The procedure was explained to the patient to ease fear and to ensure cooperation. Usually, 4-5 slides were prepared and 2-3 slides were instantly inserted into a fixative jar for wet fixation. Others were air-dried for May-Grunwald-Giemsa stain (MGG) and acid-fast bacilli (AFB). The data obtained were coded and entered into Microsoft Excel Worksheet (Microsoft® Corp., Redmond, WA, USA). The study data were analyzed using SPSS software for Windows version 22 (IBM Corp., Armonk, NY, USA).

## Results

Out of 50 patients, the majority (34%) belong to the 31-40 age range, with a mean age of 38.29 ± 10.77 years. Compared to men (26%), women comprised the majority of patients (74%). These findings suggest that the age range of 31-40 is the most frequently affected group among the patients included in the study. Furthermore, it highlights the higher prevalence of women seeking medical attention for the condition compared to men. This information can be valuable in understanding the demographics and potential risk factors associated with the particular disease being studied. Table 1 shows demographic insights from patient data.

**Table 1:** Demographic insights from patient data: Age range and gender distribution

Variables		No. of Patients (n=50)	Percentage
Age group (years)	≤20	03	06.00
	21-30	12	24.00
	31-40	17	34.00
	41-50	08	16.00
	>50	10	20.00
Gender	Female	37	74.00
	Male	13	26.00

Table 2 shows the symptoms reported by 50 patients experiencing thyroid swelling. All patients (100%) reported swelling over the neck, making it the most common complaint. Pain was reported by 26% of the patients, followed by pressure effects of 18%. Sixteen percent of the individuals observed loudness of the voice, while 8% of the patients reported both dysphagia and pyrexia each. These findings indicate that while neck swelling was universally present, other symptoms such as pain, pressure effects, and changes in voice or difficulty swallowing were also notably reported among a subset of patients.

**Table 2:** Clinical symptoms of thyroid swelling in patients

Complaints	Number of Patients	Percentage (%)
Swelling over neck	50	100.00
Pain	13	26.00
Pressure Effects	09	18.00
Hoarseness of Voice	08	16.00
Dysphagia	04	8.00
Pyrexia	04	8.00

Table 3 shows the clinical diagnoses observed in a group of 50 patients with thyroid conditions. The most common diagnosis was a single thyroid nodule, present in 27 patients, comprising 54% of the cases. Subsequently, diffuse thyroid swelling was observed in 15 patients, representing 30% of the cases. Multinodular goiter and cystic lesions were less frequent, each observed in four patients, representing 8% of the cases individually. These diagnoses reflect the varied presentations of thyroid conditions within the studied patient cohort, with solitary nodules being the most prevalent among the observed cases.

**Table 3:** Clinical diagnosis in patients

Clinical Diagnosis	Number of Patients	Percentage (%)
Solitary thyroid nodule	27	54.00
Diffuse thyroid swelling	15	30.00
Multi-nodular goiter	04	08.00
Cystic lesion	04	08.00

Table 4 presents the distribution of diagnoses among 50 patients with thyroid conditions. Most, comprising 68%, were diagnosed with colloid goiter, followed by multinodular colloid goiter at 18%. Thyroiditis was observed in 8% of the cases, while a smaller percentage was attributed to malignancies, with papillary carcinoma diagnosed in 4% of patients and anaplastic carcinoma noted in 2%.

**Table 4:** Distribution by FNAC diagnosis of patients with thyroid conditions. FNAC - Fine needle aspiration cytology

Diagnosis	Number of Patients	Percentage
Colloid goiter	34	68.00
Multi-nodular colloid goiter	09	18.00
Thyroiditis	04	08.00
Papillary carcinoma	02	04.00
Anaplastic carcinoma	01	02.00
Total	50	100

Table 5 compares the classification of lesions diagnosed by FNAC with those determined by histopathology in a sample of 50 cases. It reveals that FNAC identified 47 cases (94%) as benign lesions, closely with the 43 cases (86%) determined as benign by histopathology. However, FNAC identified a smaller number, three cases (6%), as malignant, while histopathology found seven cases (14%) as malignant. The total number of cases, both in FNAC and histopathology, reaches 50 (100%), highlighting the distribution of benign and malignant lesions as diagnosed by these respective methods.

**Table 5:** Comparison of FNAC with histopathology diagnosis FNAC - Fine needle aspiration cytology

Classification	FNAC (%)	Histopathology (%)
Benign lesions	47 (94.00)	43 (86.00)
Malignant	03 (6.00)	07 (14.00)
Total	50 (100)	50 (100)

Table 6 presents the performance metrics of FNAC as a diagnostic tool for thyroid nodules. It indicates that FNAC demonstrates a high sensitivity of 95.38%, suggesting its effectiveness in correctly identifying patients with thyroid nodules, with a low chance of false negatives. However, the specificity is relatively lower at 53.33%, indicating a higher probability of false positive results, which could lead to misdiagnosis of patients without thyroid nodules. The positive predictive value (PPV) stands at 86.67%, indicating that among those diagnosed positive for thyroid nodules by FNAC, there is a high likelihood that they genuinely have the condition. The negative predictive value (NPV) is 88.57%, indicating that patients who test negative for thyroid nodules using FNAC are highly likely to be free of the condition, providing reassurance and potentially avoiding unnecessary follow-up tests.

**Table 6:** Sensitivity and specificity of FNAC for thyroid nodules FNAC - Fine needle aspiration cytology; PPV - Positive predictive value; NPV - Negative predictive value

FNAC	Value	95% CI
Sensitivity	95.38%	91.06% to 99.96%
Specificity	53.33%	34.90% to 92.21%
PPV	86.67%	66.43% to 97.16%
NPV	88.57%	60.60% to 98.74%

## Discussion

The majority of patients were found to be in the age groups of 31 to 40 years (34%), subsequently by the age group of 21 to 30 years (24%), according to the current study. Women made up the bulk of the patients (74%), while only 26% were men. Middle-aged adults are more prone to the condition being studied, while young adults also seem to be at risk. The overwhelming majority of patients being women raises questions about potential gender-specific factors that may contribute to the development of the condition. More research should investigate the reasons behind these age and gender disparities to improve prevention and treatment strategies. Identical results were found in studies by Rout et al. [6], Bhise et al. [7], and Kumar et al. [8], in which the mean age of the patients was, 36 ± 13 years, 38.32 ± 11.23 years, and 37 ± 12 years, respectively. These studies provide evidence that the average age of patients in this population is around 37 years old, with a standard deviation ranging from 11.23 to 13 years. This suggests that most patients fall within the range of 24 to 50 years of age. These findings highlight the relatively young age distribution of patients in this particular context. Gupta et al. [9] found that the highest incidences (51%) of thyroid enlargements were found in the age group of 21-40 years, with females predominating (77%). This suggests that thyroid enlargements may be more prevalent in younger adults, particularly among women. Rathod et al. [10] stated that most of the patients (35%) were in the 30-39 age group, the youngest being 12 years old and the oldest 65 years old. Seventy percent of the patients were women and 30% were male.

Clinical complaints indicated that swelling and discomfort were the two concerns (26%). Identical results were observed in studies by Bhise et al. [7] and Kumar et al. [8], which found that all patients had the clinical complaint of swelling. The most common finding was solitary thyroid nodules (54%), followed by widespread thyroid enlargement (30%), multinodular goiters (8%), and cystic lesions (8%). Other less common findings included thyroiditis (4%) and hyperthyroidism (2%). Most patients with solitary nodules underwent a fine needle biopsy to determine if the nodule was benign or malignant [11]. Surprisingly, only a small percentage of nodules were found to be cancerous (6%), reassuring most patients [12]. Identical outcomes were observed in the studies by Bhise et al. [7] and Kumar et al. [8], where the clinical diagnosis of diffuse thyroid swelling was established among patients in 38% and 32%, respectively.

In 94% of cases, benign lesions were found, whereas malignant lesions were present in 6% of the cases. An FNAC sensitivity of 95.38%, a specificity of 53.33%, a positive predictive value of 86.67% and a negative predictive value of 88.57% were recorded for benign lesions, showing that FNAC is an efficient method to detect these lesions. Similar findings were observed in the study by Bhise et al. [7] and Kumar et al. [8], where the sensitivity of FNAC was observed among 91% and 83.3% of the patients, respectively. The consistency of FNAC thyroid swelling diagnosis in this series was 96.05% [13]. Comparable results were also observed in the study by Handa et al. [14], and Esmaili and Taghipour [15]. Basharat et al. [16] observed that FNAC with histopathology, sensitivity, specificity, positive predictive value, negative predictive value, and diagnostic precision was 80%, 97.7%, 80%, 97.7%, and 96%, respectively. The specificity in this study may be low due to the smaller number of cases in the study.

The study sample size is relatively small, involving only 50 patients from a specific region and within a limited timeframe. This could limit the generalizability of the findings to broader populations. Second, while FNAC demonstrates high sensitivity to detect thyroid nodules, the specificity remains relatively low, suggesting a notable chance of false positive results. This discrepancy raises concerns about the precision of diagnosing patients without thyroid nodules, which can lead to unnecessary procedures. Furthermore, the study emphasizes FNAC as a primary diagnostic tool despite acknowledging its limitations, emphasizing the need for further improvement in diagnostic accuracy. Lastly, research underscores the importance of FNAC, but acknowledges that histopathology remains the gold standard, implying that FNAC may not entirely replace histopathology in diagnosing thyroid swellings.

Limitations

The study's limitations primarily stem from its small sample size and the regional specificity of the patient cohort, which may affect the generalizability of the findings to broader populations. Additionally, the relatively low specificity of FNAC suggests potential for false positive diagnoses, indicating a need for cautious interpretation of results in the absence of confirmatory histopathological analysis.

## Conclusions

FNAC is a simple, economical, and commonly used first-line diagnostic method for thyroid cancer. A false negative or false positive cytological diagnosis may be produced as a result of thyroid cytology. The thyroid cytological results should be cross-examined with clinical symptoms and TFT and ultrasound data to avoid a false negative or false positive diagnosis. Establishing a preoperative diagnosis of thyroid FNAC is critical to determine its diagnostic value. Histopathology is the gold standard for the diagnosis of thyroid disease. The study emphasizes the need to improve basic healthcare in rural India by treating FNAC as a first-line diagnostic test for thyroid swellings to guide management, although it does not replace histopathology.
